# An in vitro model of the macrophage-endothelial interface to characterize CAR T-cell induced cytokine storm

**DOI:** 10.1038/s41598-023-46114-y

**Published:** 2023-11-01

**Authors:** Robert S. Rosen, Jason H. Yang, Juan S. Peña, Rene Schloss, Martin L. Yarmush

**Affiliations:** 1 Department of Biomedical Engineering, Rutgers University, Piscataway, NJ USA; 2 Rutgers Robert Wood Johnson Medical School, Piscataway, NJ USA; 3 Center for Emerging and Re-Emerging Pathogens, Rutgers New Jersey Medical School, Newark, NJ USA; 4 Department of Microbiology, Biochemistry and Molecular Genetics, Rutgers New Jersey Medical School, Newark, NJ USA; 5 Center for Engineering in Medicine, Massachusetts General Hospital, Boston, MA USA

**Keywords:** Inflammatory diseases, Acute inflammation, Cell therapies, Cancer immunotherapy, Adverse effects

## Abstract

Chimeric Antigen Receptor (CAR) T-cell therapy is a highly effective treatment for B-cell malignancies but limited in use due to clinically significant hyperinflammatory toxicities. Understanding the pathophysiologic mechanisms which mediate these toxicities can help identify novel management strategies. Here we report a novel in vitro model of the macrophage-endothelial interface to study the effects of CAR T-cell-induced cytokine storm. Using this model, we demonstrate that macrophage-mediated inflammation is regulated by endothelial cell activity. Furthermore, endothelial inflammation occurs independently of macrophages following exposure to CAR T-cell products and the induced endothelial inflammation potentiates macrophage-mediated inflammatory signaling, leading to a hyperinflammatory environment. While corticosteroids, the current gold standard of care, attenuate the resulting macrophage inflammatory signaling, the endothelial activity remains refractory to this treatment strategy. Utilizing a network model, coupled to in vitro secretion profiling, we identified STAT3 programming as critical in regulating this endothelial behavior. Lastly, we demonstrate how targeting STAT3 activity can abrogate endothelial inflammation and attenuate this otherwise hyperinflammatory environment. Our results demonstrate that endothelial cells play a central role in the pathophysiology of CAR T-cell toxicities and targeting the mechanisms driving the endothelial response can guide future clinical management.

## Introduction

Chimeric Antigen Receptor (CAR) T-Cell therapy has revolutionized the treatment and management of patients with relapsing and refractory B-cell malignancies, showing complete remission in up to 80% of its recipients^1^. Despite its clinical success, CAR T-cell therapy carries severe ‘black-box warning’ hyperinflammatory toxicities associated with its administration, particularly a Cytokine Release Syndrome (CRS) and Immune Effector Cell Associated Neurotoxicity Syndrome (ICANS), which are highly prevalent in recipients of this drug. CRS occurs in over 90% of patients, and ICANS in up to 60% of patients, and management of these side-effects is often at great cost to the patients and healthcare system^2–7^. Understanding how these hyperinflammatory syndromes develop can reduce the burden of these toxicities, drive down costs, and facilitate the use of this otherwise life-saving therapy to a wider patient population.

Preclinical studies of CAR T-cell toxicities have transformed our understanding of the pathophysiology and subsequent clinical management. CRS was originally hypothesized to be a direct result of T-cell mediated inflammation; however, in vivo models demonstrated that the origins of CRS reside in macrophages^8–11^. Thus, clinical management has focused on regulating macrophage inflammatory products through direct cytokine inhibition or corticosteroids. Yet, both CRS and ICANS can remain refractory to this management strategy^10, 12^ suggesting an alternative mechanism beyond macrophage origins alone. Recent clinical evidence suggests the vascular endothelium plays a significant role in the development of these hyperinflammatory conditions, particularly the neurotoxicity, whose prevalence is highly correlated with markers of endothelial dysfunction^13–17^. Clinical emergence of endothelial dysfunction, characterized by increased pro-inflammatory signaling (e.g., IL-6, IL-8, MCP-1), loss of barrier function, coagulopathy, etc., is highly correlated with CAR T-cell dose and subsequent severity of the toxicities^18–20^. Endothelial dysfunction is also highly correlated with macrophage-derived systemic inflammatory markers descriptive of CRS suggesting the endothelium contributes to its pathogenesis as well^2, 21^. Despite this growing body of evidence, the underlying pathophysiologic mechanisms leading to endothelial dysfunction, its contribution to the hyperinflammatory environment, and the interplay of the vascular endothelium with inflammatory macrophages, has yet to be explored.

In the present studies, we develop and characterize a novel multicellular in vitro model of the soluble macrophage-endothelial interface following CAR T-cell therapy to begin to elucidate the pathophysiologic role of the vascular endothelium in CAR T-cell toxicities. The model herein described recapitulates key in vivo corollary behaviors to these clinical toxicities to manifest individual cellular contributions to their pathogenesis. Using this model, we demonstrate that the interaction of macrophages and endothelial cells manifests in distinct responses unseen in monoculture conditions, and this interaction influences the inflammatory response. We identified STAT3 activity as a potential mechanism regulating the endothelial behavior and demonstrated how modulating its activity can help regulate the observed inflammation. Collectively, the data presented here situate endothelial cells as central players in the development of CAR T-cell toxicities and interventions designed to protect the endothelium may help ensure safe and efficacious delivery of current and future CAR T-cell products to a wider patient population.

## Results

### Macrophage-endothelial crosstalk mediates inflammatory signaling

Macrophages are known to be the major inflammatory cell type mediating CRS^9, 22^ so we hypothesized that pro-inflammatory macrophages would induce endothelial activation in hyperinflammatory conditions. Macrophages alter their secretion profiles in response to environmental cues, classically represented by the M1-M2 axis, denoting pro- or anti-inflammatory polarizations, respectively^23^, and lipopolysaccharide (LPS) stimulation is the prototypical M1/pro-inflammatory polarizing agent. While there is no consensus for what constitutes a ‘cytokine storm’^24^, LPS-mediated hyperinflammation induces pro-inflammatory signaling in macrophages akin to CRS^25^ and as such served as an initial model, and subsequent positive control, for hyperinflammation.

**Figure 1:**
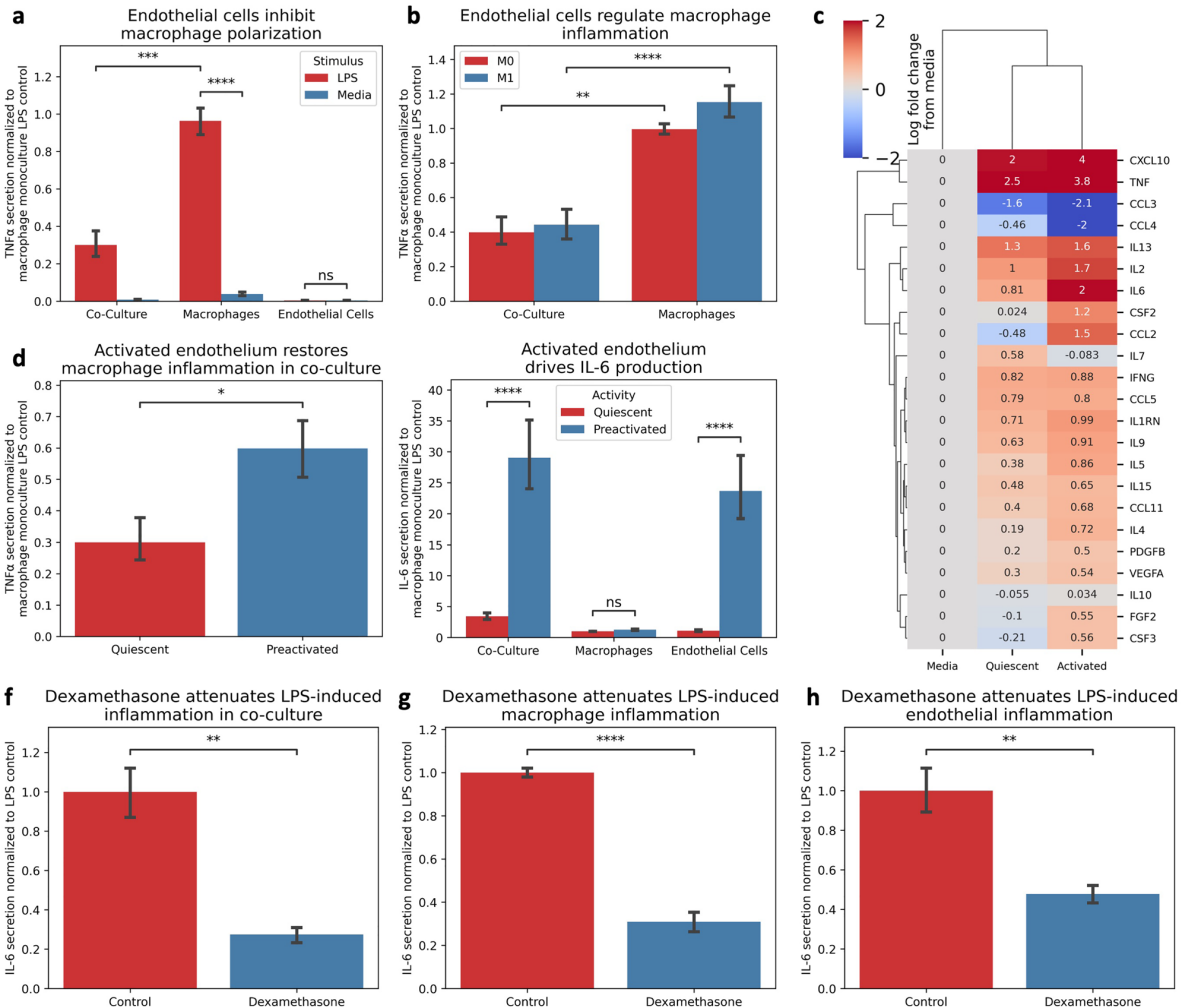
Endothelial Activation Influences Macrophage Pro-Inflammatory Signaling. (**A**) The presence of endothelial cells in co-culture attenuate macrophage TNF secretion compared to monoculture control following 1 ug/mL LPS stimulation. (**B**) Endothelial cells regulation of macrophage TNF is agnostic to pre-existing macrophage phenotype. (**C**) increased endothelial activity influences macrophage inflammatory secretion profile. (**D**) Increased endothelial activity increases macrophage TNF secretion compared to quiescent endothelial conditions. (**E**) Endothelial cells drive IL-6 secretion compared to macrophage secretion profile. (**F**) Dexamethasone (8 ug/mL) attenuates LPS-Inflammation in Co-Culture. G. Dexamethasone (8 ug/mL) attenuates LPS-Inflammation in macrophage monoculture. H. Dexamethasone (8 ug/mL) attenuates LPS-Inflammation in endothelial monoculture. * Indicates *p* < 0.05, ** indicates *p* < 0.01, *** indicates *p* < 0.001, **** indicates *p* < 0.0001.

We developed a co-culture system of the soluble macrophage-endothelial interface (Supplemental Fig. 1E) to understand the crosstalk between macrophages and endothelial cells and challenged it to LPS stimulation. Surprisingly, we found that macrophage TNFα secretion in co-culture was attenuated in the presence of quiescent endothelial cells (Fig. 1A, Supplemental Fig. 1F) compared to the monoculture control, regardless of the pre-existing macrophage phenotype (Fig. 1B), suggesting a regulatory role of vascular endothelial cells on macrophage pro-inflammatory programming.

Given the blunted macrophage secretion profile, we hypothesized that endothelial activity might regulate the inflammatory response, and inflammatory endothelial activity would facilitate macrophage inflammation. We primed endothelial cells with inflammatory cytokines, integrated them with our macrophage model, and then challenged the system to LPS-stimulation. We observed a restoration of pro-inflammatory secretions (Fig. 1C), increasing macrophage-derived cytokine release (e.g., TNFα, Fig. 1D), and an increase in systemic inflammatory marker IL-6 driven by the endothelial cells (Fig. 1E), indicating the endothelial activity influences macrophage inflammatory signaling and contributes to the hyperinflammatory environment. Notably, LPS-mediated inflammation was well controlled following treatment with dexamethasone in both mono- and co-culture conditions for both cell types (Figs. 1F–H). Collectively, these data demonstrate that macrophage inflammation is influenced by crosstalk with endothelial cells, highlighting the importance of this cellular interaction, and the context-dependent regulation of their inflammatory response.

### CAR T-cells directly induce endothelial dysfunction

While LPS-stimulation provides a strong initial model for hyperinflammation, it may not capture the true pathophysiology. Onset of toxicities follow CAR T-cell engagement with the tumor associated antigen and subsequent T-cell inflammation and cytotoxic activity, so we next sought to characterize the endothelial state following CAR T-cell treatment.

**Figure 2:**
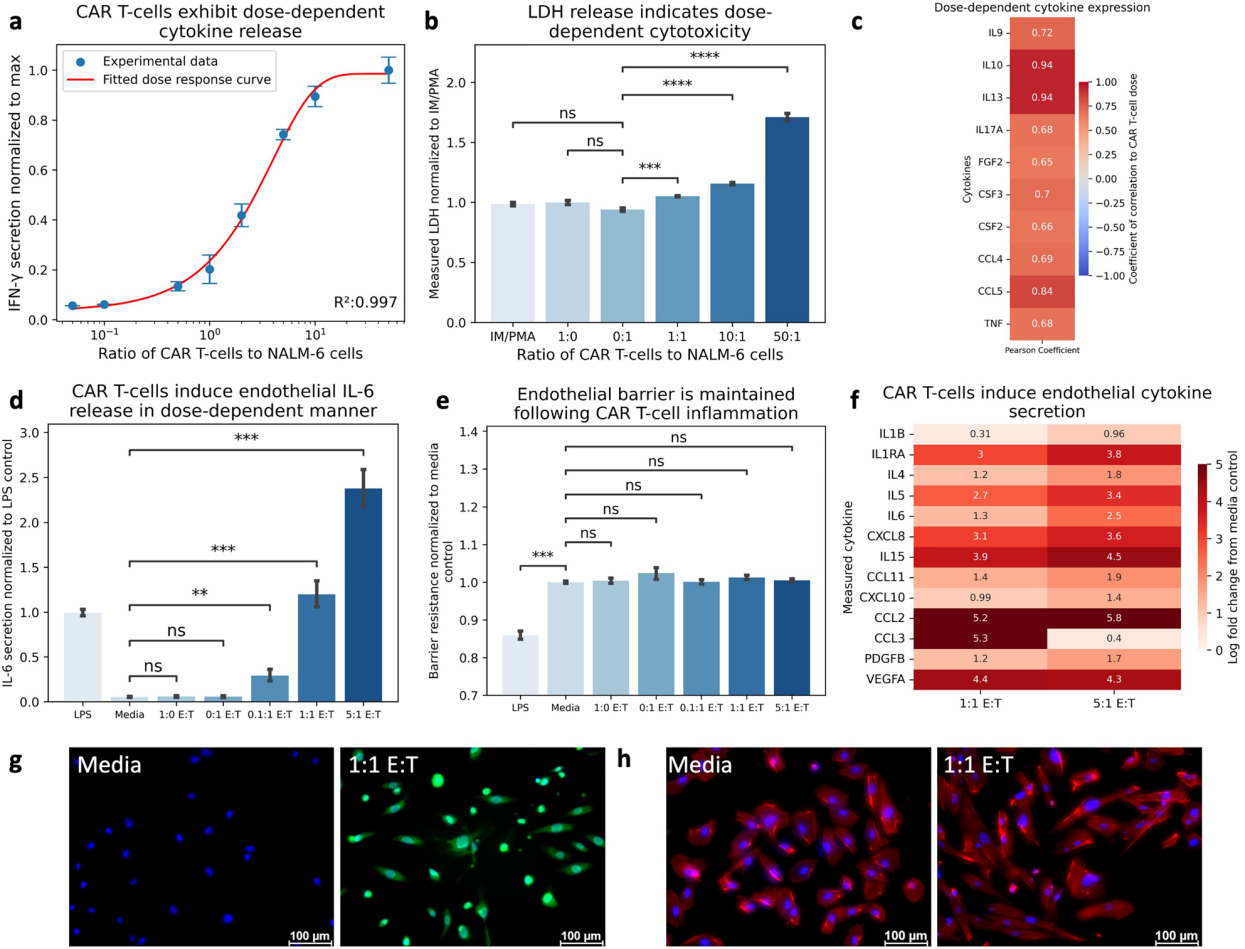
CAR T-cell inflammation Induces Dose-Dependent Endothelial Response. (**A**) Co-Culture of CAR T-cells and NALM6 cells display dose-dependent increase in IFN-γ release. (**B**) Co-Culture of CAR T-cells and NALM6 cells results in dose-dependent release of Lactate Dehydrogenase indicated dose-dependent cytotoxicity. (**C**) Pearson Correlation of cytokine secretion to ratio of CAR T-cells to NALM6 cells indicate dose-dependent cytokine release. (**D**) Activated CAR T-cell conditioned media induces IL-6 secretion from endothelial cells in dose-dependent manner. (**E**) Activated CAR T-cell conditioned media does not alter endothelial barrier resistance. (**F**) Endothelial cells exhibit unique cytokine release in dose-dependent manner to CAR T-cell conditioned media. (**G**) CAR T-cell conditioned media induces endothelial ROS expression (Blue = DAPI, Green = CellRox). (**H**) CAR T-induced inflammation results in cytoskeletal rearrangement (Blue = DAPI, Red = F-Actin). * Indicates *p* < 0.05, ** indicates *p* < 0.01, *** indicates *p* < 0.001, **** indicates *p* < 0.0001. E:T = Effector to Target ratio, the ratio of CAR T-cells to NALM6 cells.

We co-cultured primary 1928z CAR T-cells with their target tumor associated antigen cells, CD19^+^ NALM6 cells. T-cell engagement with its target antigen produced a dose-dependent increase in IFN-γ (Fig. 2A), and dose-dependent expression of characteristic T-cell derived cytokines (Fig. 2C). T-cell engagement also resulted in a dose-dependent release of Lactate Dehydrogenase (LDH), indicating cellular cytotoxicity (Fig. 2B). Notably, activated CAR T-cells did not produce IL-6, the hallmark biomarker of both CRS and ICANS. Collectively, these findings are consistent with previous reports of CAR T-cell inflammation and cytotoxic function^8^.

Since endothelial activity influences macrophage pro-inflammatory signaling, we first sought to characterize the endothelial response to CAR T-cell therapy. We cultured 1928z CAR T-cells with their target tumor associated antigen presenting cells at varying effector-to-target ratios (i.e., a dose–response) and exposed endothelial monoculture to the cell-free conditioned media. Endothelial cells developed an inflammatory phenotype in response to CAR T-cell conditioned media, resulting in a dose-dependent increase in IL-6 production (Fig. 2D), as well as with multiple other cytokines and chemokines otherwise not expressed from CAR T-cells directly (Fig. 2F). The endothelium demonstrated upregulation of reactive oxygen species (ROS) in response to these stimuli (Fig. 2G). Interestingly, endothelial monolayers did not alter their barrier resistance in response to activated CAR T-cell conditioned media (Fig. 2E), although they did exhibit increased polarity and morphological changes indicated through actin cytoskeletal rearrangement through endothelial elongation (Fig. 2H, Supplemental Fig. 2A). These findings suggest that endothelial inflammation partially occurs due to CAR T-cell signaling, however, this only induced some, but not all, of the pathophysiologic features descriptive of these toxicities.

### Macrophage-endothelial crosstalk manifests unique pathophysiologic features

**Figure 3:**
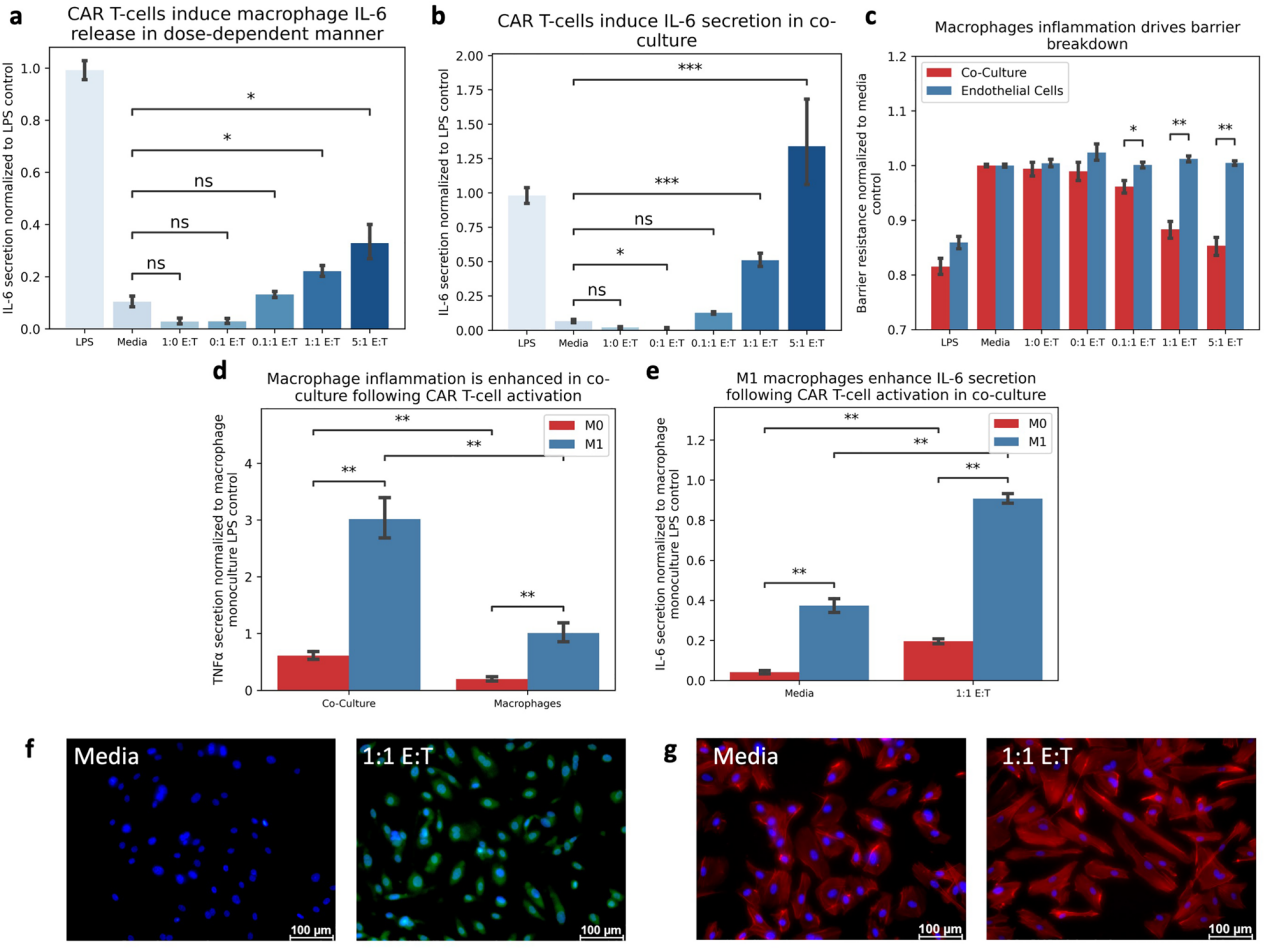
CAR T-Induced Endothelial Activity Promotes Macrophage Inflammation. (**A**) Macrophages respond to CAR T-cell conditioned media by increasing IL-6 secretion in dose-dependent manner. (**B**) Co-culture system increases IL-6 secretion in dose-dependent manner. (**C**) Co-culture of endothelial cells and macrophages displays decreased endothelial barrier resistance correlating with CAR T-cell dose. (**D**) Co-Culture macrophages increase TNF secretion following treatment with CAR T-cell conditioned media compared to monoculture controls. (**E**) M1 macrophages enhance IL-6 secretion in monoculture compared to unpolarized control following treatment with CAR T-cell conditioned media. (**F**) Endothelial ROS production increases in Co-Culture following treatment with CAR T-cell conditioned media (Blue = DAPI, Green = CellRox). (**G**) CAR T-induced inflammation results in cytoskeletal rearrangement in co-culture (Blue = DAPI, Red = F-Actin). * Indicates *p* < 0.05, ** indicates *p* < 0.01, *** indicates *p* < 0.001, **** indicates *p* < 0.0001. (E:T = Effector-to-Target ratio).

Macrophages are considered the primary inflammatory cell mediating CRS^9, 22^ and our macrophage monoculture model exhibited dose-dependent IL-6 secretion and other cytokines such as IL1 in response to CAR T-cell conditioned media (Fig. 3A, Supplemental Fig. 4), consistent with these reports. Since the endothelium is directly activated through CAR T-cell mediated inflammation and macrophage activity can be influenced by endothelial activity, we hypothesized that crosstalk between these cell types would amplify macrophage cytokine release following exposure to CAR T-cell conditioned media. We challenged our co-culture system to CAR T-cell conditioned media and found a dose-dependent increase in IL-6 secretion (Fig. 3B), consistent with monoculture controls. Notably, co-culture conditions, and subsequent crosstalk between endothelial cells and macrophages, yielded a dose-dependent breakdown in the endothelial barrier otherwise unseen in endothelial monoculture controls (Fig. 3C), consistent with the dose-dependent increase in endothelial activity and its effect on macrophage activation. In fact, the co-culture system increased expression of multiple cytokines including TNFα compared to monoculture control (Fig. 3D, Supplemental Fig. 5), further indicating that CAR T-mediated endothelial activity amplified macrophage activity. Additionally, M1 macrophages maintained their polarization, resulting in a significant increase in TNFα (Fig. 3D) and IL-6 (Fig. 3E) levels compared to unpolarized controls, further driving CRS presentation. The endothelium continued to express increased ROS (Fig. 3F) and display an altered cytoskeletal structure (Fig. 3G), consistent with monoculture controls, indicating altered endothelial activity following CAR T-cell conditioned media treatment. Collectively, this data suggests that interplay of these target cell types contributing to disease development, simultaneously driving macrophage and endothelial inflammation, and results in unique cellular behaviors otherwise unseen in monoculture.

### Endothelial inflammation is refractory to current standard of care

**Figure 4:**
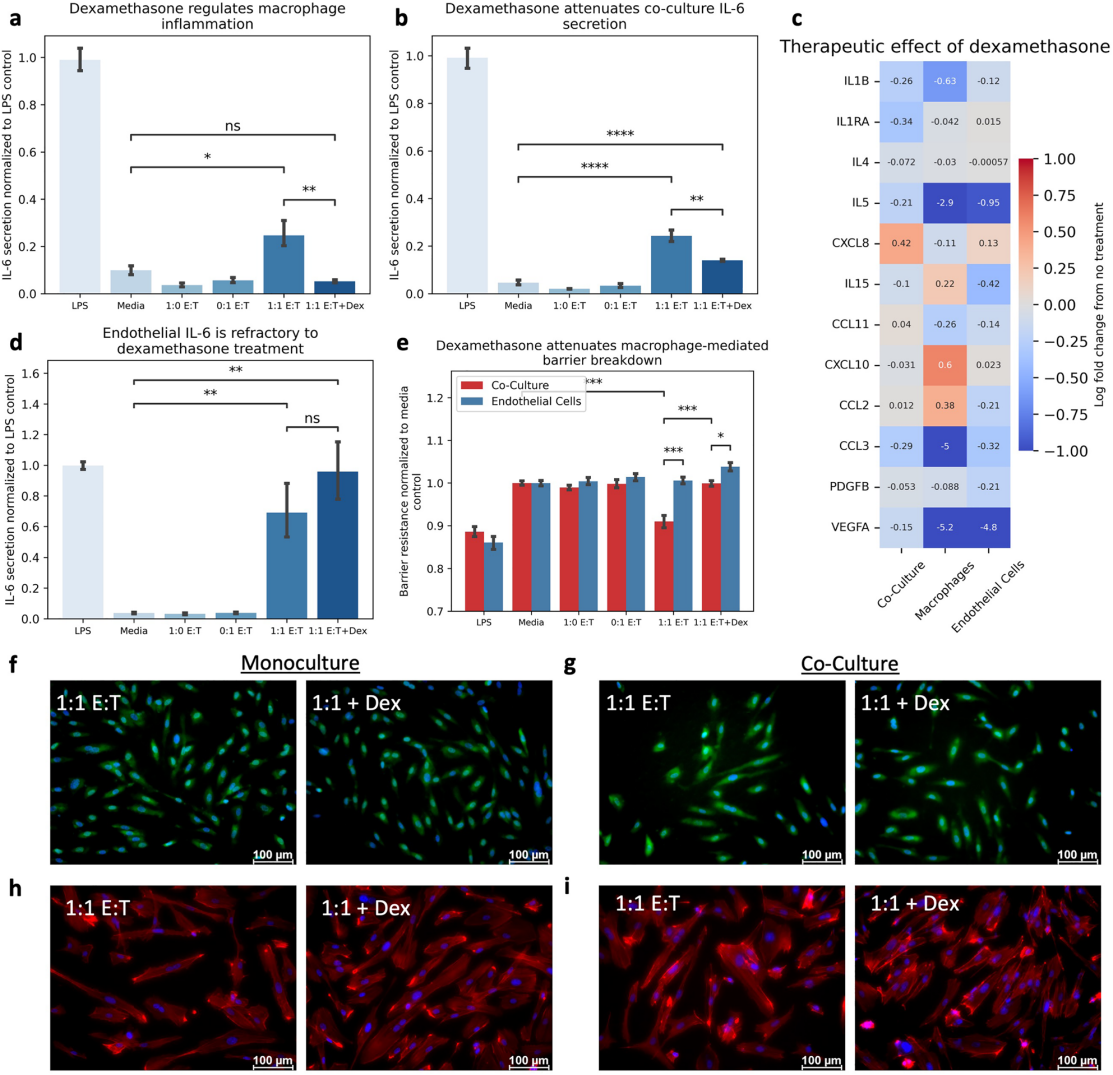
Endothelial Inflammation is Refractory to Dexamethasone Treatment. (**A**) Dexamethasone attenuates macrophage-mediated IL-6 production induced by CAR T-cell conditioned media. (**B**) Dexamethasone attenuates IL-6 production in co-culture system following CAR T-cell conditioned media treatment. (**C**) Log fold change in cytokine secretion following dexamethasone treatment of CAR T-cell conditioned media by culture system. (**D**) Dexamethasone does not alter endothelial cell IL-6 production following treatment with CAR T-cell conditioned media. (**E**) Dexamethasone increases endothelial barrier resistance in co-culture. (**F**) Endothelial ROS production in monoculture is refractory to dexamethasone treatment (Blue = DAPI, Green = CellRox). (**G**) Endothelial ROS production in co-culture is refractory to dexamethasone treatment (Blue = DAPI, Green = CellRox). (**H**) Dexamethasone does not alter endothelial morphology in monoculture (Blue = DAPI, Red = F-Actin). (**I**) Dexamethasone does not alter endothelial morphology in co-culture (Blue = DAPI, Red = F-Actin). * Indicates *p* < 0.05, ** indicates *p* < 0.01, *** indicates *p* < 0.001, **** indicates *p* < 0.0001. (E:T = Effector-to-Target ratio).

Clinical management of severe CAR T-cell toxicities utilize corticosteroids, where grading of severity is based on both clinical presentation and lab values (e.g. IL-6)^26^. We challenged our co-culture and monoculture systems to treatment with dexamethasone to investigate its therapeutic efficacy on IL-6 secretion. Dexamethasone attenuated macrophage IL-6 secretion (Fig. 4A), consistent with the clinical efficacy observed against CRS. This regulation of IL-6 production was also notable in co-culture, although to a lesser extent (Fig. 4B). Critically, endothelial IL-6 secretion exposed to CAR T-cell conditioned media in monoculture remained refractory to dexamethasone treatment (Fig. 4D), suggesting that dexamethasone is ineffective against CAR T-cell induced endothelial inflammation. In fact, dexamethasone primarily attenuated macrophage-derived cytokines, such as IL-1 and CXCL8, but had little effect on endothelial-driven cytokine production directly (Fig. 4C). Dexamethasone did restore the endothelial barrier function in co-culture (Fig. 4E), further supporting its efficacy on the macrophage-derived contribution to the pathophysiology. Yet, endothelial ROS expression (Fig. 4F-G) and morphologic changes indicated through cellular elongation (Fig. 4H-I) were unaltered by dexamethasone treatment, regardless of the culture system, suggesting the direct endothelial activity was unattenuated. Collectively these data suggest that dexamethasone is efficacious for reducing macrophage inflammation but has limited efficacy against the endothelial response following CAR T-cell mediated inflammation.

### Network analysis identifies STAT3 is critical to endothelial inflammation

**Figure 5:**
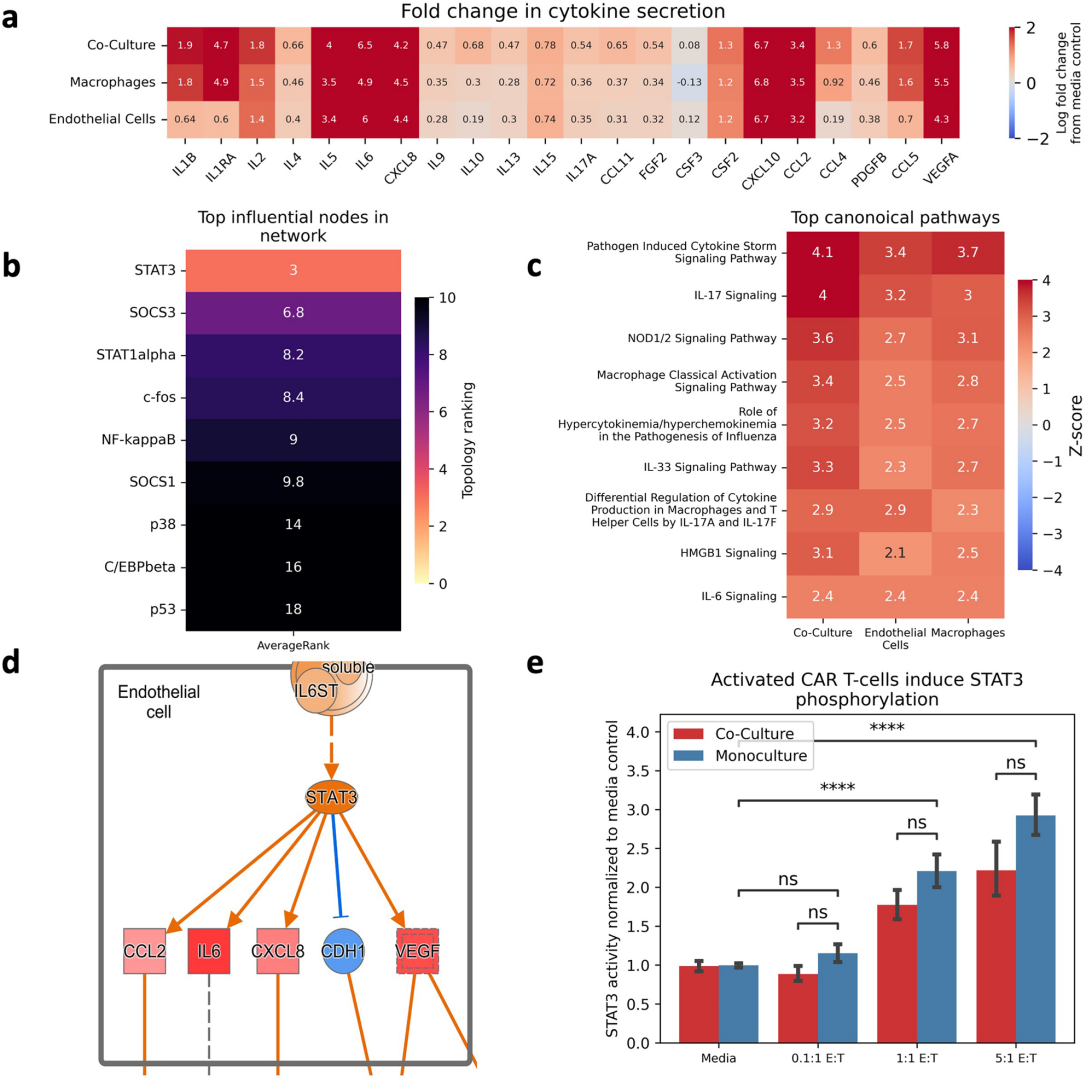
Network Analysis Identifies Potential Axis of Endothelial Inflammation. (**A**) Multiplex evaluation of culture cytokine secretion profiles represented as log fold change from media baseline. (**B**) Heatmap ranking average topological rankings of each node/protein in the endothelial network. (**C**) Ranking of IPA canonical pathways enriched in each culture system. (**D**) Mapped multiplex data to Cytokine Storm pathway predicts STAT3 activity is increased in culture system correlating with CAR T-cell dose. Image provided with permission from Ingenuity Pathway Analysis by Qiagen. (**E**) STAT3 activity increases following CAR T-cell treatment in vitro. * Indicates *p* < 0.05, ** indicates *p* < 0.01, *** indicates *p* < 0.001, **** indicates *p* < 0.0001.

The anti-inflammatory effect of dexamethasone is mediated through inhibition of NF-kB activity ^27, 28^, so we hypothesized that endothelial activation occurred through an NF-kB independent mechanism. To identify a potential pathway mediating the observed endothelial changes following exposure to CAR T-cell products, we coupled together cell-specific computational network models, pathway enrichment analysis, and our in vitro observations. We pruned prior knowledge network databases for pathways connecting known inflammatory mediators of CRS and ICANS^18^, traversing signal transduction pathways through ligands, receptors, secondary messengers, and transcription factors. We then filtered this inflammatory signaling network by cell-specific expression data to generate cell-specific signal transduction networks. We evaluated the secretion profile of each culture system (Fig. 5A) to generate corollary in vitro data for each cell-specific network. Initial evaluation of these networks for influential mediators identified endothelial-STAT3 as highly ranked across all topology metrics (Fig. 5B), suggesting that STAT3 may play a role in this system. Culture secretion profiles were then analyzed using Ingenuity Pathway Analysis. The top enriched pathway across all culture systems was “Pathogen Induced Cytokine Storm Signaling Pathway” (Fig. 5C), and mapping of these secretion profiles to this pathway further suggested STAT3 activity as a potential mechanism mediating this endothelial expression profile (Fig. 5D). Based on these findings, we hypothesized that endothelial-STAT3 activity increased following exposure to CAR T-cell conditioned media and blocking its activity would ameliorate the altered expression patterns observed in our culture system. We measured the activity of endothelial-STAT3 in our culture system and found a dose-dependent, and notably, macrophage-independent increase following treatment with CAR T-cell conditioned media (Fig. 5E). Collectively, this data suggests that STAT3-mediated programming is associated with endothelial dysfunction following exposure to CAR T-cell secretion.

### Regulating STAT3 attenuates endothelial response

**Figure 6:**
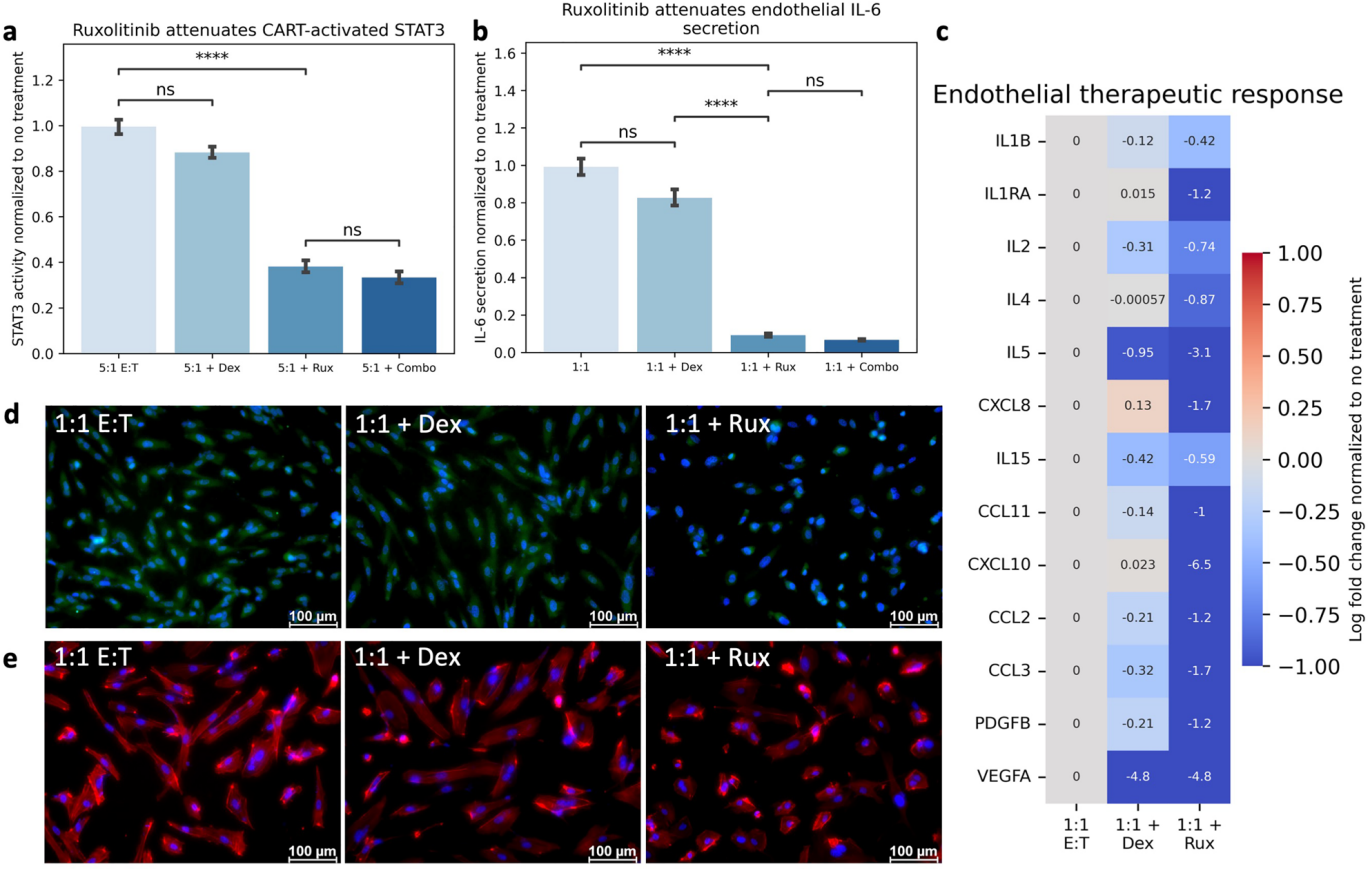
Endothelial Dysfunction is Regulated Through STAT3 Programming. (**A**) Ruxolitinib treatment attenuates CAR T-cell induced endothelial STAT3. (**B**) Ruxolitinib attenuates Endothelial IL-6 Secretion. (**C**) Ruxolitinib attenuates endothelial cytokine release. (**D**) Ruxolitinib Reduces Endothelial ROS Expression (Blue = DAPI, Green = ROS). (**E**) Ruxolitinib Restores Endothelial Morphology (Blue = DAPI, Red = F-Actin). * Indicates *p* < 0.05, ** indicates *p* < 0.01, *** indicates *p* < 0.001, **** indicates *p* < 0.0001. 1:1 E:T = effector to target ratio of CAR T-cells to NALM6 cells. Dex = Dexamethasone (8 ug/mL). Rux = Ruxolitinib (20 uM).

To discern whether the observed dose dependent increase in STAT3 activity regulates this endothelial response, we inhibited STAT3 activity using ruxolitinib. Ruxolitinib, but not dexamethasone, attenuated the endothelial STAT3 activity following CAR T-cell inflammation (Fig. 6A) and modulation of this transcription factor led to a significant reduction in endothelial IL-6 production following treatment with CAR T-cell conditioned media (Fig. 6B). This reduction was seen across multiple endothelial-derived cytokines, supporting a key role of STAT3 activity in regulating the endothelial secretory response (Fig. 6C). Notably, ruxolitinib treatment also attenuated endothelial ROS expression (Fig. 6D) and cytoskeletal rearrangement (Fig. 6E, Supplemental Fig. 2). Collectively, this data suggests that the observed endothelial dysfunction following CAR T-cell treatment is at least partially regulated through STAT3 programming.

### Regulating the macrophage-endothelial interface attenuates CAR T-cell toxicities

**Figure 7:**
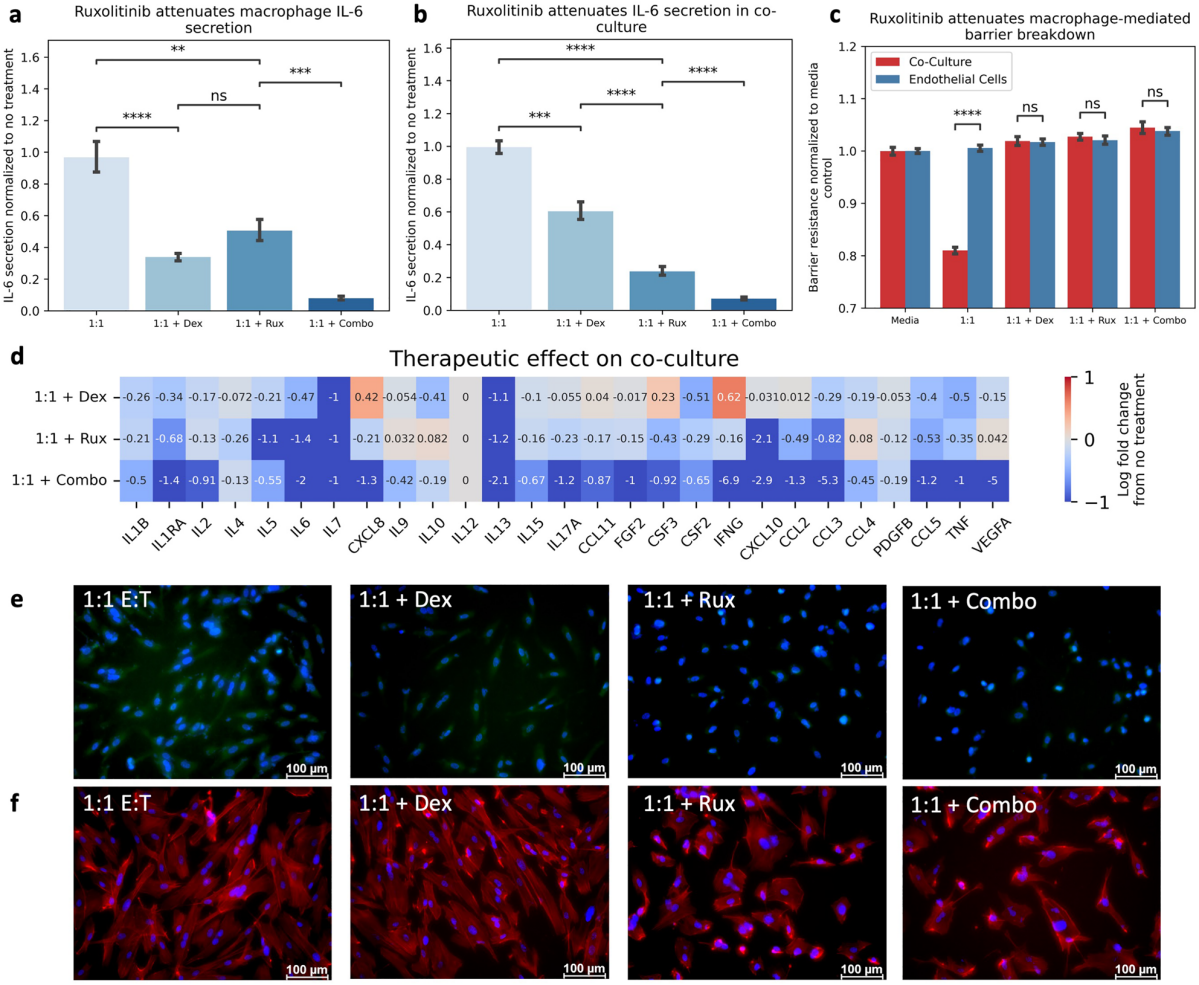
Combination Therapy Attenuates Markers of CAR T-cell Toxicities. (**A**) Treatment of macrophage monoculture with dexamethasone (Dex), ruxolitinib (Rux) or combination (combo) resulted in decreased IL-6 secretion. (**B**) Treatment of co-culture with dex and rux resulted in decreased IL-6 secretion. (**C**) All treatments restore endothelial barrier resistance. (**D**) Combination therapy reduced fold change in cytokine release greater than individual treatments alone. (**E**) Ruxolitinib, but not dexamethasone, reduces endothelial ROS Expression (Blue = DAPI, Green = CellRox). (**F**) Ruxolitinib reduced endothelial cytoskeletal rearrangement (Blue = DAPI, Red = F-Actin). * Indicates *p* < 0.05, ** indicates *p* < 0.01, *** indicates *p* < 0.001, **** indicates *p* < 0.0001. (1:1 = effector to target ratio of CAR T-cells to NALM6 cells). Dex = Dexamethasone. Rux = Ruxolitinib.

Crosstalk in the co-culture system manifests unique features otherwise unseen in monoculture conditions due to activity of both cell types. We challenged our model to treatment targeting each cell type, using dexamethasone for macrophages, ruxolitinib for endothelial cells, or a combination of the two, to discern whether regulating each component directly may yield a greater therapeutic benefit. Both dexamethasone and ruxolitinib attenuated macrophage-derived IL-6 production, and this effect was amplified in combinatorial treatment (Fig. 7A), suggesting that STAT3 activity, in addition to NF-kB activity, may contribute to the CAR T-cell induced IL-6 response in macrophages. When integrated with endothelial cells, combinatorial treatment yielded the greatest attenuation of IL-6 production (Fig. 7B). Barrier integrity was restored following all treatments (Fig. 7C). Combination treatment compared to individual treatments exhibited the greatest reduction across numerous pro-inflammatory cytokines (Fig. 7D). The effect of ruxolitinib directly on the endothelium was consistent with monoculture controls, demonstrating decreased ROS expression (Fig. 7E) and restoration of actin rearrangement (Fig. 7F). Collectively, these data suggest that targeting both endothelial dysfunction and macrophage inflammation can reduce the observed markers of this pathophysiology.

## Discussion

CAR T-cell therapy is a highly effective cancer therapy with life-threatening, hyperinflammatory toxicities. Understanding how this treatment induces these toxicities may assist in facilitating the design of safe and efficacious products. Here we described a novel in vitro system to study the interaction of macrophages and endothelial cells following CAR T-cell treatment to recapitulate the key pathophysiologic features of CAR T-cell mediated sequalae and begin to elucidate the specific role of each cell type in its pathophysiology. Our model demonstrated the importance of this cellular interaction following CAR T-cell administration, particularly in the regulation of hyperinflammatory phenotypes. Current risk mitigation strategies for these toxicities focus on regulating macrophage inflammatory products and non-specific immunosuppressive treatments, which may be ineffective and potentially detrimental to CAR T-cell efficacy.

The endothelium exhibited direct regulation of the macrophage inflammatory response and therefore may exacerbate CRS. Our initial studies in an LPS model demonstrated the regulatory role of the endothelium on macrophage inflammation and this regulation was sustained regardless of the initial macrophage state. Following CAR T-cell mediated inflammation, and resulting endothelial activation, macrophage inflammation was enhanced in the presence of endothelial cells compared to monoculture controls. This resulted in an increase in macrophage inflammatory products, including IL-1 and IL-6, as well as other cytokines and chemokines (Supplemental Fig. 5). Whether this macrophage inflammation is initiated due to direct CAR T-cell stimulation or through the endothelial regulation of macrophages remains to be elucidated. Critically, M1 macrophages sustained their activation and increased levels of both TNF and IL-6, suggesting a potential inflammatory amplification loop due to direct endothelial activation. The mechanism through which this regulation occurs has yet to be determined, but one possible explanation is the significant increase in GM-CSF released by stimulated endothelial cells (Supplemental Fig. 3), which is known to enhance macrophage inflammatory products in reaction to CAR T-cell inflammation^22, 29^. Efforts have been made to engineer CAR T-cells to reduce their secretion of GM-CSF as a method of reducing toxicities since activated CAR T-cells secrete GM-CSF/CSF3 in a dose-dependent manner (Fig. 2C), however, if an alternative source contributes to overall GM-CSF, then this approach may not be an effective strategy.

Many cytokines have been reported as biomarkers of this pathophysiologic state, and peak serum levels occur in various phases^25, 30^. Early expression of characteristic T-cell derived cytokines such as IL-2, IL-7, IL-15, and GM-CSF occur following CAR T-cell administration and are associated with severity of ICANS^18^. Elevation and peak serum expression of macrophage-derived cytokines soon follow, including IL-1, IL-6, CXCL8/IL-8, TNF, and IL-10. While there are many cytokines implicated in this disease process, their significance to its pathogenesis can vary greatly, however IL-6 has emerged as a central cytokine in this syndrome. Here we highlighted the contribution of macrophages and endothelial cells towards the production of IL-6, and the production of numerous other cytokines and chemokines (Supplemental Figs. 4, 5). CAR T-cell induced expression of endothelial-derived CCL2, CCL3, and CXCL8, chemokines involved in macrophage and leukocyte chemotaxis and inflammation, may provide a potential mechanism by which the endothelium aids in this pathophysiology. The contribution of chemokine-mediated recruitment of inflammatory macrophages due to direct endothelial activation warrants further investigation.

The endothelial dysfunction is often framed as a secondary effect of the macrophage inflammatory response to CAR T-cells; however, our data suggests that endothelial inflammation is a direct result from CAR T-cell therapy. Endothelial cells increased ROS expression and altered their cytoskeletal structure in response to CAR T-cell conditioned media and dexamethasone demonstrated poor efficacy in mediating these endothelial activity markers, highlighting the limitations of this therapeutic modality. ROS signals downstream inflammation and coagulation^31^, and its expression in endothelial cells is associated with blood–brain barrier dysfunction and neuroinflammation^32, 33^. The actin cytoskeleton plays a crucial role in the expression of junctional proteins, surface molecule expression, and the interaction with circulating leukocytes and diapedesis^34, 35^. In response to CAR T-cell conditioned media, endothelial cells demonstrated loss of their cortical actin rim and formation of stress fibers, which is known to increase gaps between cells, enabling passage of cytokines, chemokines, and cells across the endothelial barrier^36, 37^. Furthermore, the endothelium expressed multiple chemotactic agents following CAR T-cell conditioned media treatment which may facilitate leukocyte extravasation. In fact, extravascular presence of CAR T-cells has been observed in the CSF of ICANS patients^2^, further suggesting the effects of direct endothelial activation should not be discounted in the pathogenesis of CAR T-cell toxicities, particularly ICANS.

STAT3 activity in endothelial cells has been shown to regulate vascular barrier function^38^ and endothelial inflammation, and regulating STAT3 activity has shown promising results in maintaining vascular integrity^39^. Aberrant STAT3 signaling has become implicated in many other pathophysiologic states clinically analogous to CRS and ICANS, including sepsis^39, 40^, viral-induced cytokine storm^41–47^, as well as other diseases^48^. Notably, dysfunction in this pathway has also been implicated in leukemia and lymphoma patient populations due to intrinsic lymphocytic cytokine signaling^49^, and following administration of chemotherapy preconditioning regimens such as fludarabine^50^, a clinical predictor of CAR T-cell toxicity severity^51^. Several studies targeting this signaling cascade for mediating CRS toxicity have undergone investigation in clinical trials using ruxolitinib^52^. These studies have demonstrated positive results for management of steroid-refractory CRS, consistent with our observed effect on macrophage inflammation. However, studies have also shown that non-specific JAK inhibition may dampen the efficacy of CAR T-cells and this effect must therefore be considered before clinical implementation^53, 54^. Yet, this dampening effect delays CAR T-cell expansion, but does not completely inhibit tumor clearance capabilities, suggesting that the timing of this therapy may be key to maximizing therapeutic efficacy. It should also be noted that while JAK inhibition has been studies in the context of CRS^12, 53–55^, it has yet to be explored for the indication of ICANS which is primarily driven by endothelial dysfunction^2, 21^. Future clinical trials should investigate efficacy towards neurotoxicity as a primary outcome.

THP-1 macrophages have long been established as representative models of macrophage physiology. Our macrophage model demonstrates robust phenotypic plasticity (Supplemental Fig. 1A,B) consistent with our previous work in primary macrophages^23^ and has also been consistent with previously reported in vitro systems of CAR T-cell toxicities. One report utilized primary monocytes in co-culture with CAR T-cells and their target tumor antigen to find an increase in myeloid-derived IL-6 secretion^22^. In response to CAR T-cell conditioned media, the macrophage monoculture system increased IL-6 and IL-1 secretion, as well as other cytokines and chemokines implicated in CRS (Fig. 3A, Supplemental Fig. 4), consistent with these previous reports^9, 10, 22^. Yet, THP-1 macrophages may not fully recreate the function of primary cells, nor the magnitude of response to inflammatory cues^56, 57^. Future work will aim to incorporate primary cell sources to better capture the pathophysiology. We selected to use HUVECs as an initial model, since it is already well characterized for vascular function and inflammation, and our initial studies were consistent with these previous in vitro models (Supplemental Fig. 1C,D)^58^. A previously reported in vitro model of CAR T-cell toxicities also identified direct endothelial activation in HUVECs in response to CAR T-cell and macrophage inflammation^59^, but did not explore the feedback effect onto the macrophage population. It is important to recognize that endothelial cells also display heterogenous phenotypes across the vasculature and HUVECs only represent one phenotypic subtype. Future studies should be conducted to investigate the role of individual endothelial subtypes, which have been shown to demonstrate great heterogeneity in their function and morphology, particularly brain microvascular cells and their role in the development of ICANS.

The model herein described offers key insights into CAR T-cell toxicities. While originally thought to be of macrophage origins, this model demonstrates that endothelial activity, and its effect on macrophages, is critical towards understanding these toxicities. This model system enabled the interrogation of this macrophage-endothelial interface and the dissection of their individual cellular contributions towards these toxicities. While the focus of this work is on hyperinflammatory environments, this model can be easily utilized to understand other macrophage-endothelial mediated disease processes, such as with foam cells in cardiovascular disease, giant cells in vasculopathies, or tumor associated macrophages in solid malignancies. Furthermore, this approach is adaptable to incorporate patient-derived cell sources, which can facilitate identifying patient-specific responses and unique genomic risk factors associated with this already personalized therapeutic.

## Conclusion

Hyperinflammatory toxicities following treatment with CAR T-cell therapy pose a serious bottleneck in accessibility to this highly effective cancer treatment. Understanding how these toxicities develop is critical to developing novel risk mitigation strategies for the safe delivery of these biologic products. The data presented in these studies demonstrate that increased endothelial activity, accompanied by endothelial-STAT3 programming, emerges independently of macrophages following CAR T-cell therapy, and is refractory to the current standard of care alone. Crosstalk between these activated endothelial cells and macrophages enables amplification of their inflammatory secretion profiles and the manifestation of unique cellular behaviors, characteristic of CAR T-cell toxicities. Therefore, the development and implementation of endothelial-protective therapeutic strategies may ultimately improve the overall safety of CAR T-cell products and facilitate their use in the clinical setting.

## Materials and methods

### Cell culture & media composition

THP-1 (ATCC) and NALM-6 (ATCC) cells were maintained and activated in complete media consisting of RPMI 1640 media with 10% Fetal Bovine Serum (FBS), 1% penicillin and streptomycin, and 55 mM of beta-mercaptoethanol and incubated in a humidified atmosphere at 37C with 5% CO_2_. Human Umbilical Vein Endothelial Cells (HUVECs) were maintained in M200 Media supplemented with Low Serum Growth Supplement (LSGS) or equivalent growth factors and 1% penicillin and streptomycin. Primary human 1928z Chimeric Antigen Receptor (CAR) T-cells were gifted from Century Therapeutics. CAR T-cells were cultures in advanced RPMI 1640 media supplemented with 10% FBS, 1% Pen/Strep, 1% GlutaMAX, and 55 mM beta-mercaptoethanol. Media was supplemented every 2–3 days and cells are passaged every 5–6 days to maintain a cellular density below 1e6 cells/mL. All media components were acquired from Thermofisher, unless stated otherwise.

### Culture model set up & conditions

#### CAR T-cell activation

CAR T-cells were co-cultured with their target antigen cell, NALM-6 cells, at the designated effector-to-target ratio at a base density of 1 × 10^5^ cells/mL (i.e., 1:1 is 1 × 10^5^ CAR T-cells to 1 × 10^5^ NALM6 cells). Following 24 h of incubation, the media was centrifuged to remove cells and debris, and the conditioned media was used to stimulate the mono- and co-culture models or analyzed directly. CAR T-cell activation was confirmed with IL-2 and IFN-γ ELISAs (Biolegend) and Lactate Dehydrogenase Cytotoxicity Assay (Invitrogen).

#### Mono- and co-culture models

Three days prior to experiments, 1.5 × 10^5^ cells/well of THP-1 monocytes were differentiated to macrophages using 16 nM of PMA (Sigma Aldrich), as previously described^23^. Macrophages were allowed to attach to the plate, and after 24 h, the differentiation media was replaced with fresh THP-1 media for an additional 48 h. One day prior to the experiment, HUVECs were trypsinized, centrifuged, and resuspended in HUVEC Base Media, and 20,000 cells/well were plated in Corning 24-well plate Transwells (0.4 um) to adhere overnight. On the day of the experiments, all media was removed, and endothelial transwells were integrated with the macrophage culture.

#### Culture conditions

For all experiments, fresh media supplemented with 1 ug/mL LPS (Sigma Aldrich) and fresh media without any supplementation was used as a positive and negative control, respectively. Endothelial pre-activation was conducted by incubating endothelial cells for three hours with 20 ng/mL IL-1β (Peprotech) prior to integration into co-culture system. Macrophage polarization was conducted by incubating macrophages for three hours with 1 ug/mL Lipopolysaccharide (Sigma Aldrich) for M1 populations, or media for M0, prior to integration into co-culture system. Dexamethasone (R&D Systems) was supplemented at a concentration of 8 ug/mL, unless indicated otherwise. Ruxolitinib (R&D Systems) was supplemented at a concentration of 20 uM, unless indicated otherwise. Vehicle controls of DMSO or PBS at equivalent volumes were conducted for all experiments (data not shown). All data presented represents conditions after 24 h of incubation.

### Staining & imaging

Endothelial cells were stained with CellRox Green (Invitrogen) for reactive oxygen species expression following the manufacturer’s protocol. Cells were washed thrice with PBS, counterstained with Hoeschst (0.2 uL per mL) for 10 min to visualize cell nuclei, fixed with 4% paraformaldehyde, and imaged within 3 h of fixation. Cells were stained with Phalloidin 594 (Invitrogen) at 1:400 for 1 h at room temperature to visualize polymerized actin structure. All images generated were acquired on an Olympus IX81 microscope or Leica microscope.

### Secretion profiling

All cytokine measurements were obtained from cell culture supernatants using Biolegend ELISA Max Plates or a Human Inflammatory Cytokine Multiplex Bead Assay of up to 27 cytokines (Bio-Rad) as per manufacturers’ instructions. A full list of cytokines measured by Human Inflammatory Cytokine Multiplex Bead Assay can be found in the Table 1. STAT3 and Phosphorylated STAT3 were measured using STAT3 (pY705 + Total) ELISA Kit (Abcam). Supernatants and cell lysates collected from each condition were stored at -80C prior to analysis. For each measurement, the supernatants were thawed, centrifuged to remove debris, and diluted in the provided buffer to concentrations necessary to fall within the reference range of the ELISA standard curve.

**Table 1:** Measured Cytokines.

FGF basic	IFN-γ	IL-4	IL-8	IL-13	MCP-1	RANTES
Eotaxin	IL-1β	IL-5	IL-9	IL-15	MIP-1a	TNF-a
G-CSF	IL-1ra	IL-6	IL-10	IL-17	MIP-1b	VEGF
GM-CSF	IL-2	IL-7	IL-12 (p70)	IP-10	PDGF-BB	

### Trans-endothelial electrical resistance

Trans-Endothelial Electrical Resistance (TEER) was assessed using an EVOM2 voltmeter (World Precision Instruments) per manufacturer’s instructions. All data represents changes in resistance values normalized to media control.

### Network generation and topology

An initial cellular-agnostic network was generated by combining previously reported network models of macrophage and endothelial phenotypes. Two extensive macrophage network models have been previously reported^60, 61^. Additionally, relevant models of endothelial cells have been used in migration, proliferation, angiogenesis, and junctional phenotyping and used to define barrier integrity, coagulation, and endothelial behavior^62–64^. Leveraging these existing frameworks, we combined these reported networks using Cytoscape (version 3.8.2) to form a cellular-agnostic interaction network.

This network was pruned for intermediate pathways connecting measured mediators as either inputs or outputs. The search method used a modified Depth First Search algorithm to find all simple paths, where a simple path is defined as any connection between two nodes (i.e., from the input to the output) with no repeating intermediate node, to create a pathology-specific, cellular-agnostic network. We limited the depth of this search to 8 nodes due to an exponential increase in time to form this network. To generate cell-specific networks, we filtered the network by expression data of each model cell type using RNA expression data available through the Human Protein Atlas. Cell-specific networks were then analyzed for topologic features using the NetworkX toolbox in Python. Topologic features included in-degree centrality, out-degree centrality, closeness centrality, eigenvector centrality and vote rank. All generated networks are available as simple interaction format (SIF) files in supplemental files.

### Ingenuity pathway analysis (IPA)

Multiplexed secretion profiles were analyzed using Ingenuity Pathway Analysis (IPA; Qiagen) version 01–21-03. Briefly, all ‘5:1 E:T’ secretion profiles were normalized to ‘5:1 E:T’ CAR T-cell conditioned media profiles and log transformed to represent changes from the baseline media profile. The log-transformed data was then processed through IPA core analysis, with no mutations, including direct and indirect relationships, interaction and causal networks, all node types and data sources, experimentally observed and high (predicted) confidence, and restricted to macrophage and/or endothelial cell lines. Z-scores reported represent enrichment for canonical pathways represented in this dataset, based on relationships published in the literature.

### Statistical analyses

All groups represent the mean of three independent experiments (N = 3) produced in triplicate (n = 9) normalized to their on-plate controls of either LPS or media. Comparisons within a culture system (e.g., macrophage monoculture dose–response) were normalized to cytokine measurements from the on-plate control of 1 ug/mL LPS. Comparisons between culture systems, (e.g., co-culture to macrophage monoculture TNF secretion), were normalized data to macrophage monoculture LPS control. Data presented is mean values with error bars representing the standard error of the mean. All data was initially analyzed using a Shapiro–Wilk Test for normality. Analysis of Variances (ANOVA) was used for multiple comparisons between groups with an alpha of 0.05 or non-parametric equivalent. Post-hoc analysis was conducted using a Tukey HSD test with a Bonferroni correction or non-parametric equivalent. Standard curves for quantitative measurements were constructed using four-point sigmoidal curve-fitting with a tolerance for coefficient of determination (R^2^) of 0.95.

### Supplementary Information

Supplementary Information.

## Data Availability

All data is available upon request. Please contact the corresponding author (schloss@soe.rutgers.edu) for requests for data.
