# Supplementary material for: An in vitro model of the macrophage-endothelial interface to characterize CAR T-cell induced cytokine storm
Source: Sci Rep. 2023 Nov 1;13:18835. doi: 10.1038/s41598-023-46114-y (PMC10620221; doi:10.1038/s41598-023-46114-y)
Supplement: Supplementary file 1 — Supplementary Information. [file 41598_2023_46114_MOESM1_ESM.pdf]

# Supplemental Figures

# Supplemental Figure 1

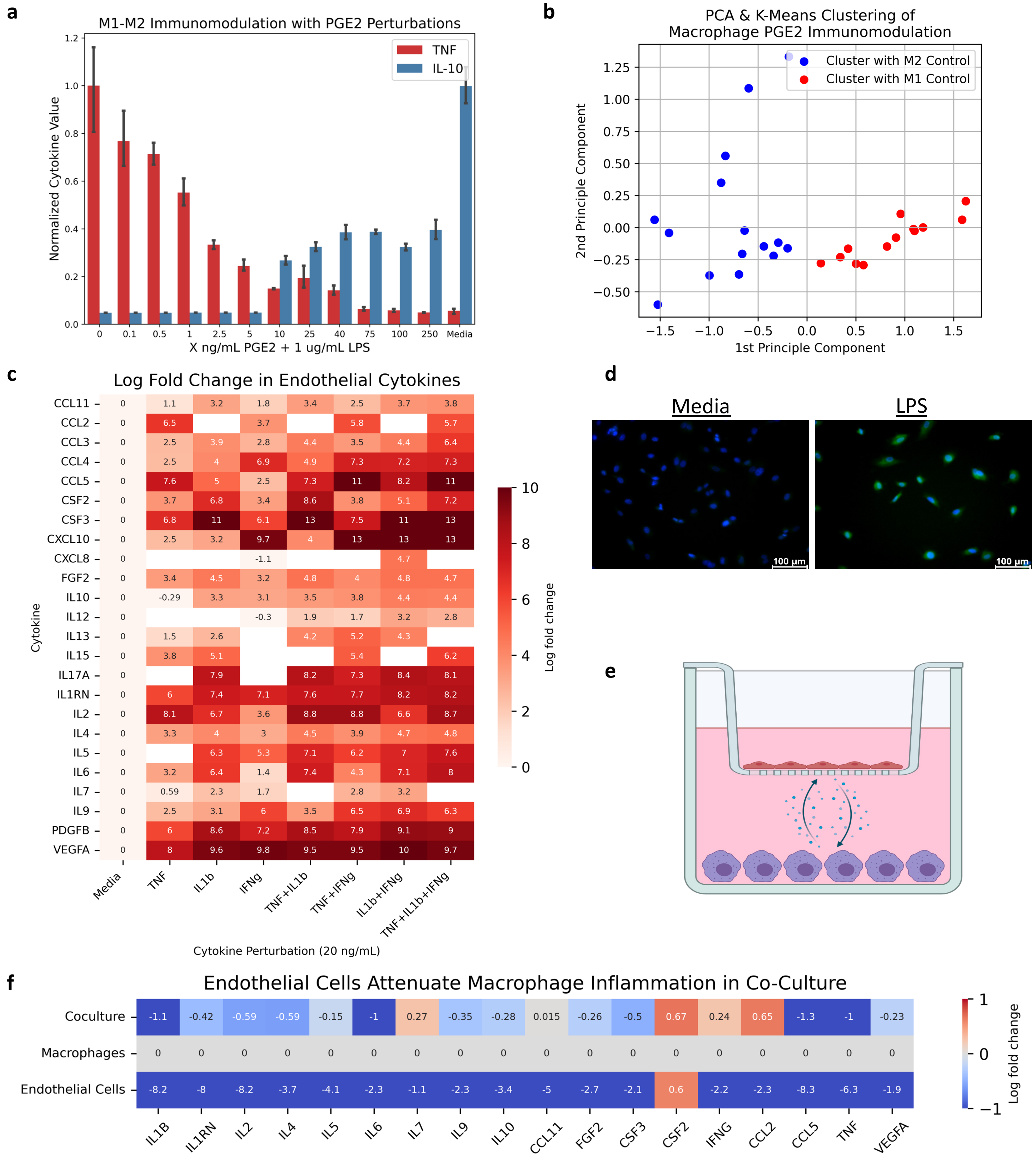


Supplemental Figure 1. A) Immunomodulation of macrophage monoculture with PGE2 dose response demonstrates attenuation of TNF secretion and promotion of IL-10 secretion, indicating transition from M1 to M2 phenotype. B) Principal component analysis following dimensional reduction of 27 cytokine multiplex analysis demonstrate regression along primary principal component and macrophage phenotypic plasticity. C) 27 cytokine multiplex secretion profile of endothelial monoculture demonstrates differential expressional activity in response to various cytokines. D) LPS induces ROS expression in endothelial cells (Blue = DAPI, Green = CellRox). E) Representation of co-culture system. F) Secretion analysis demonstrates the presence of endothelial cells attenuates macrophage inflammatory secretion following LPS stimulation. * Indicates p < 0.05, ** indicates p < 0.01, *** indicates p < 0.001, **** indicates p < 0.0001.

# Supplemental Figure 2

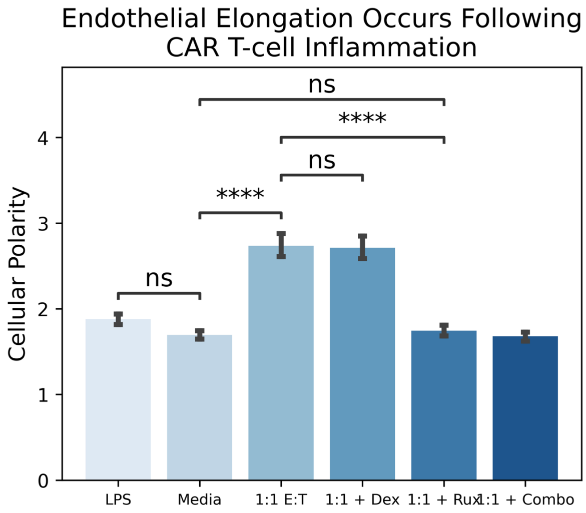


Supplemental Figure 2. Measured cellular polarity of endothelial cells in monoculture following treatment with varying conditions. * Indicates $p < 0.05$, ** indicates $p < 0.01$, *** indicates $p < 0.001$, **** indicates $p < 0.0001$. 1:1 E:T = effector to target ratio of CAR T-cells to NALM6 cells. Dex = Dexamethasone. Rux = Ruxolitinib.

# Supplemental Figure 3

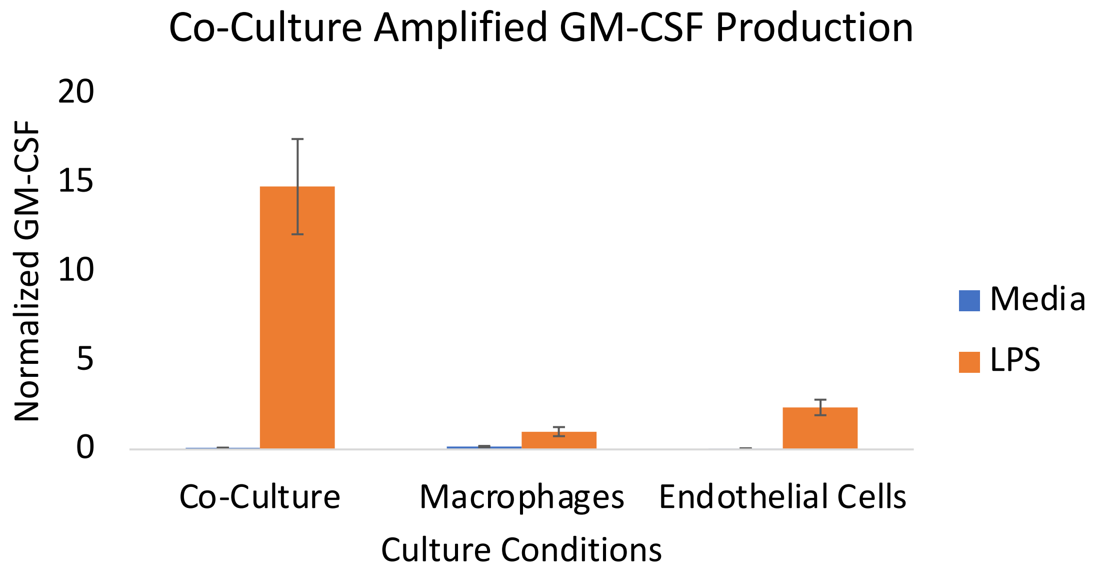


Supplemental Figure 3. Co-culture conditions enhance GM-CSF secretion following LPS stimulation. * Indicates $p < 0.05$, ** indicates $p < 0.01$, *** indicates $p < 0.001$, **** indicates $p < 0.0001$.

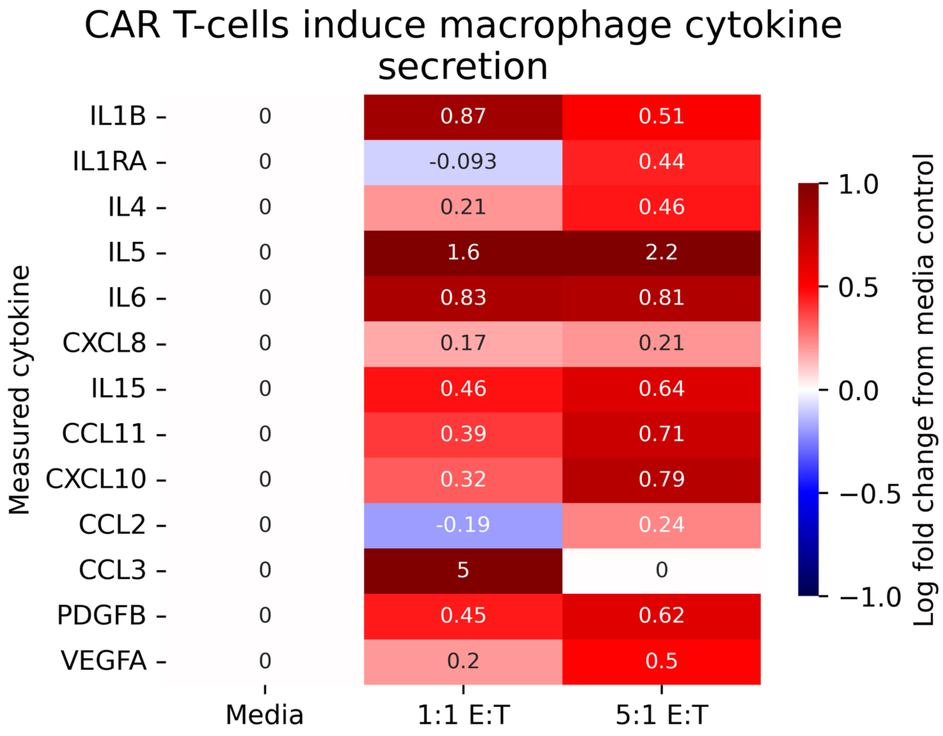


Supplemental Figure 4. Heatmap of cytokine and chemokine expression by macrophage monoculture due to different doses of CAR T-cell conditioned media compared to baseline media profile. 1:1 E:T = effector to target ratio of CAR T-cells to NALM6 cells. Data represents Log-Fold change in cytokine concentration.

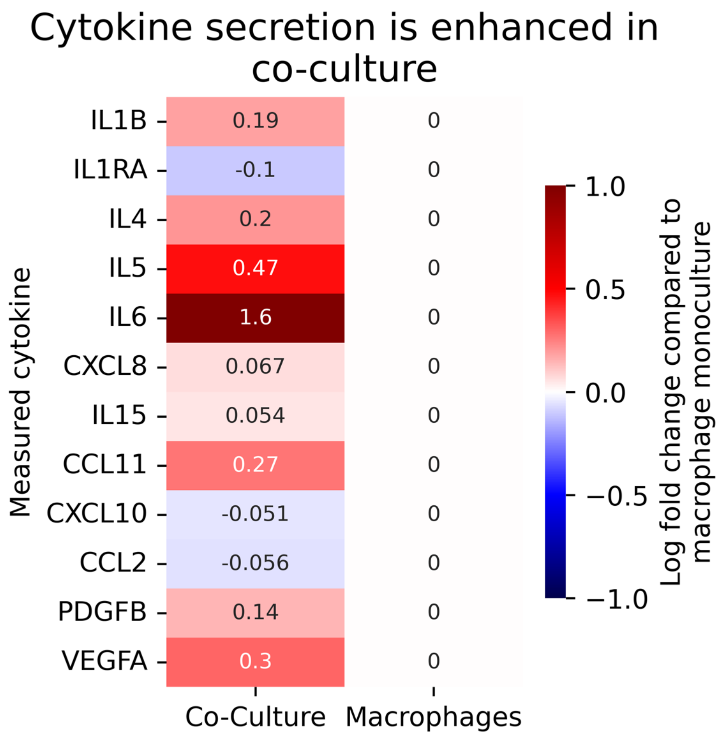


Supplemental Figure 5. Heatmap of cytokine and chemokine expression profile compared between macrophage monoculture and co-culture with endothelial cells following treatment with CAR T-cell conditioned media. Data represents Log-Fold change in cytokine concentration compared to macrophage monoculture.
